# Unraveling the complex evolutionary history of lepidopteran chromosomes through ancestral chromosome reconstruction and novel chromosome nomenclature

**DOI:** 10.1186/s12915-023-01762-4

**Published:** 2023-11-20

**Authors:** Xi Chen, Zuoqi Wang, Chaowei Zhang, Jingheng Hu, Yueqi Lu, Hang Zhou, Yang Mei, Yuyang Cong, Fangyuan Guo, Yaqin Wang, Kang He, Ying Liu, Fei Li

**Affiliations:** 1 State Key Laboratory of Rice Biology & Ministry of Agricultural and Rural Affairs Key Laboratory of Molecular Biology of Crop Pathogens and Insects, Institute of Insect Sciences, Zhejiang University, Hangzhou, China; 2 State Key Laboratory of Rice Biology, Institute of Biotechnology, Zhejiang University, Hangzhou, China; 3 Key Laboratory of Green Prevention and Control of Agricultural Transboundary Pests of Yunnan Province and Agricultural Environment/ Agriculture Environment and Resources Institute, Yunnan Academy of Agricultural Sciences, Kunming, China

**Keywords:** Lepidoptera, Sex chromosome, Comparative genomics, Sexual dimorphism, Genome evolution

## Abstract

**Background:**

Lepidoptera is one of the most species-rich animal groups, with substantial karyotype variations among species due to chromosomal rearrangements. Knowledge of the evolutionary patterns of lepidopteran chromosomes still needs to be improved.

**Results:**

Here, we used chromosome-level genome assemblies of 185 lepidopteran insects to reconstruct an ancestral reference genome and proposed a new chromosome nomenclature. Thus, we renamed over 5000 extant chromosomes with this system, revealing the historical events of chromosomal rearrangements and their features. Additionally, our findings indicate that, compared with autosomes, the Z chromosome in Lepidoptera underwent a fast loss of conserved genes, rapid acquisition of lineage-specific genes, and a low rate of gene duplication. Moreover, we presented evidence that all available 67 W chromosomes originated from a common ancestor chromosome, with four neo-W chromosomes identified, including one generated by fusion with an autosome and three derived through horizontal gene transfer. We also detected nearly 4000 inter-chromosomal gene movement events. Notably, *Geminin* is transferred from the autosome to the Z chromosome. When located on the autosome, *Geminin* shows female-biased expression, but on the Z chromosome, it exhibits male-biased expression. This contributes to the sexual dimorphism of body size in silkworms.

**Conclusions:**

Our study sheds light on the complex evolutionary history of lepidopteran chromosomes based on ancestral chromosome reconstruction and novel chromosome nomenclature.

**Supplementary Information:**

The online version contains supplementary material available at 10.1186/s12915-023-01762-4.

## Background

Lepidoptera, commonly known as butterflies and moths, represent one of the four prominent insect super-radiations, boasting approximately 160,000 cataloged species and serving crucial roles in diverse biological systems [1–3]. In addition, Lepidoptera currently has the largest number of chromosome-level genomes among animal orders [4, 5], and these species have been central to the study of chromosome evolution. Studies of the characteristics and effects of chromosome structural variation in Lepidoptera have enriched our knowledge of animal chromosome evolution [6–11]. Previous studies of three chromosome-level assemblies revealed that lepidopteran chromosomes have highly conserved synteny despite 140 million years of divergence [10–14]. However, the green-veined white butterfly (*Pieris napi*) is an exception, with its chromosome-level genome assembly showing extensive rearrangements, and the cause remains unclear [15].

Long-read DNA sequencing, optical mapping methods, and chromatin conformation capture techniques have been widely applied in whole-genome sequencing. These technologies are increasingly allowing chromosome-scale assemblies to be achieved [16]. At present, the nomenclature for newly assembled chromosomes is relatively straightforward, with the largest designated as chromosome 1 and the other chromosomes named according to the order of their size. Chromosome naming currently follows this rule in almost all species [17]. However, chromosomal rearrangements, TE insertions, and other mutation events lead to drastic changes in chromosome size [18–22], introducing inconsistencies in the size of orthologous chromosomes between species that share conserved synteny. Although these orthologous chromosomes likely originated from the same ancestral chromosome, they have different chromosome names due to changes in their size during evolution. Therefore, chromosome naming based on size may not convey information relevant to evolutionary relationships. For example, chromosome 2 of *Melitaea cinxia* does not share any synteny with chromosome 2 of *Bombyx mori* but is orthologous with *B. mori* chromosome 4 [12]. If we rename chromosome 4 of *B. mori* to chromosome 2 based on chromosome synteny with *M. cinxia*, then chromosome 2 of *M. cinxia* will be orthologous with *B. mori* chromosome 2, and evolutionary research will be much more straightforward. These discrepancies suggest that evolutionary analysis could benefit from renaming the chromosomes based on chromosome homology. For example, to facilitate research, some parrot chromosomes have been renamed based on homology [23].

Sex chromosomes are disproportionately involved in speciation and adaptation [24–29]. Lepidoptera has long served as an essential model for obtaining novel insights into sex-chromosome evolution, such as fast-Z evolution, mechanisms of dosage compensation, and the role of neo-sex chromosomes [24–33]. Prior investigations into the fast-Z evolution have primarily centered on the examination of orthologous genes, including the scrutiny of parameters such as the ratio of nonsynonymous to synonymous substitutions (dN/dS) [30, 31]. Female ancestors of Lepidoptera lacked a W chromosome with a karyotype of 30 + Z and instead utilized a ZZ/Z0 system for sex determination [34–38]. Therefore, the W chromosome of extant lepidopterans may have been acquired secondarily [34–38]. The W chromosomes were proposed to originate independently from a sex-linked B chromosome at least twice within the Lepidoptera [39–42]. Due to the dearth of previously assembled W chromosome genome sequences, W chromosome analysis is limited to a few species. Thus, their evolution and origin remain largely unknown.

Sex-linked genes tend to exhibit sex-biased expression after moving between chromosomes [43–52]. Some previous studies have focused on retrotransposon-mediated gene movement events and relied on intron loss to identify them. This method cannot identify gene movement events without intron loss. Moreover, some genes lose introns during trans-chromosomal mobilization but gain introns during evolution [5, 53]. Those studies have also ignored these intron-carrying genes, while studies using comparative genomics analysis to identify gene movements can fill this gap [43–51].

Here, we utilized 185 chromosome-level assemblies of lepidopteran insect genomes to reconstruct an ancestral reference genome of Lepidoptera. This allows us to elucidate the historical events of chromosomal rearrangements and their correlation with chromosomal features. Then, we revealed the distinctive evolutionary patterns of the Z chromosome and found evidence for a common origin of the 67 W chromosomes. We also investigated the evolutionary trajectory of inter-chromosome gene movement and their impact on phenotypes.

## Results

### Lepidopteran ancestral chromosomes and chromosomal rearrangements

**Fig. 1:**
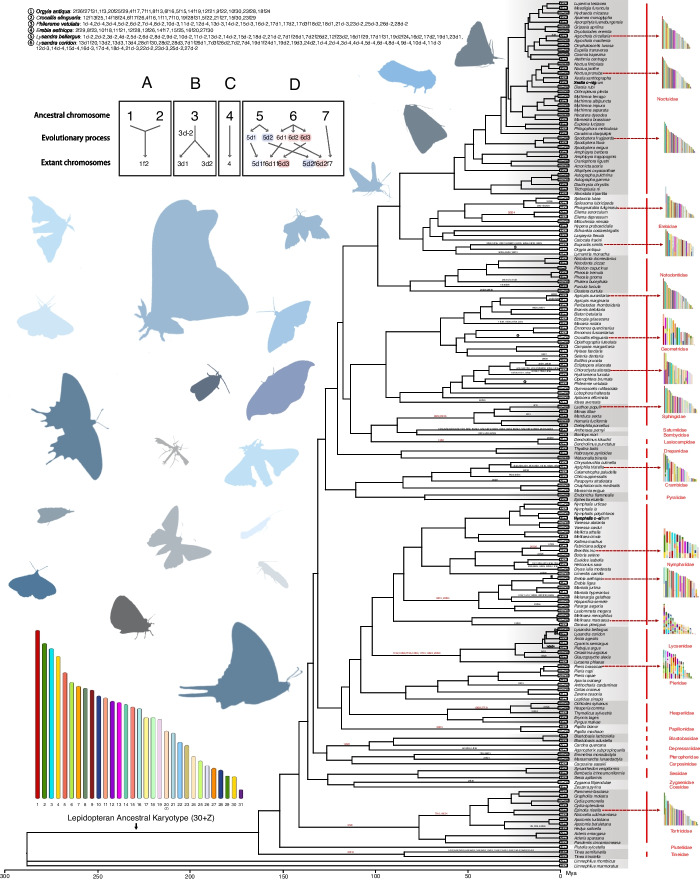
Phylogenetic tree and new chromosome nomenclature of Lepidoptera. 185 Lepidoptera from 26 families and 152 genera and two Trichoptera genome assemblies at the chromosome level were collected from InsectBase 2.0 and NCBI. These assemblies were all labeled karyotypes and had scaffold N50 > 5 Mb. We estimated a phylogeny of Lepidoptera using protein sequences of 185 Lepidoptera and two Trichoptera as outgroups. The absence of the W in the current assemblies is not an indicator of the W being absent in the species. Each fusion and fission event of 31 ancestral chromosomes was marked on the species tree with red color at ancestral levels and black color at species levels. Some with too many events to be marked on the tree are written in the upper left corner. The karyotype figures of the lepidopteran ancestor and some extant species were also shown here. Several species have not been annotated for chromosomal rearrangement events because the complexity of the chromosomal rearrangements they have experienced is beyond our current ability to resolve (see the “Methods” section). The reference ancestral genomes contained 31 chromosomes (one Z chromosome and 30 autosomes). We renamed the extant chromosomes based on their homologous with the ancestral chromosomes (Additional file 1: Tables S2 and S3). Below are examples of this nomenclature: (**A**) An extant chromosome formed by the fusion of ancestral chromosomes 1 and 2 is named 1f2. **B** The occurrence wherein ancestral chromosome 3 undergoes fission, forming two separate chromosomes, is denoted as 3d-2. Two extant chromosomes formed by the fission of ancestral chromosome 3 are named 3d1 and 3d2. **C** An extant chromosome maintained ancestral chromosome 4 without inter-chromosomal rearrangement is named 4. **D** An extant chromosome formed by the fusion of the first part of ancestral chromosome 5, the first part of ancestral chromosome 6, and the third part of ancestral chromosome 6 was named 5d1f6d1f6d3. An extant chromosome formed by the fusion of the second part of ancestral chromosome 5, the second part of ancestral chromosome 6, and ancestral chromosome 7 was named 5d2f6d2f7

To reconstruct the lepidopteran ancestral chromosomes, we downloaded the chromosome-level genome assemblies of 185 lepidopteran and two Trichopteran species from InsectBase 2.0 [5] and NCBI [54] (Additional file 1: Table S1). Among these, 70 assemblies were obtained with known annotated gene sets, and we annotated the other 117 assemblies using the BRAKER2 pipeline [55] (Additional file 1: Table S1). OrthoFinder v2.3.14 [56] with Diamond v0.8.25 [57] was used to cluster proteins into orthogroups. We then generated a time-calibrated phylogeny of Lepidoptera with the Trichopteran species serving as outgroups (Fig. 1).

Orthogroups with excessive copies will interfere with reconstructing reference ancestral genomes [58]. Therefore, we used the phylogenomic tree and the orthogroups with no more than two copies in any of the 187 species to reconstruct the lepidopteran ancestral chromosomes using AGORA [58]. The ancestral reference genome contained 31 chromosomes (Fig. 1, one Z chromosome, and 30 autosomes with a ZZ/Z0 system for sex determination in agreement with previous studies [34–38]) with 3961 ancestral genes. The ancestral chromosomes were named according to length (number of genes), with the longest ancestor chromosome containing 259 genes and the shortest harboring 14 genes. Based on its homology with the Z chromosome in extant species, ancestor chromosome 20 was identified as the ancestor of the Z chromosome, which contains 104 genes (Fig. 1).

If an extant chromosome and an ancestral chromosome shared at least eight orthologous genes, they were considered orthologous chromosomes (see the “Methods” and “Discussion” sections). Following this standard, orthologous ancestral chromosomes were identified for each extant chromosome. These 31 ancestral chromosomes appeared to serve as 31 essential elements that formed the diverse karyotypes of different lepidopteran species through fusion and fission [10–14] (Additional file 1: Tables S2 and S3), similar to Muller elements in *Drosophila* and Nigon elements in nematodes [59–62].

If two extant chromosomes were found to be orthologous with the same ancestral chromosome, by extension, these chromosomes were also considered orthologous and expected to have conserved synteny between them. To test the reliability of the reconstructed ancestral chromosomes, we used MCScanX to conduct all vs. all whole-genome synteny analysis among the 187 species. The orthologous relationships between chromosomes identified by synteny analysis were consistent with those revealed by ancestral chromosome reconstruction, thus supporting the robustness of the ancestral chromosomes. Moreover, the synteny analysis showed that the number of collinear genes between any two lepidopteran species was greater than 7307 (Additional file 2: Fig. S1). These synteny analysis results, in conjunction with the species tree, suggested that while rearrangement between chromosomes is common, chromosomal synteny has been widely conserved among lepidopterans beginning at least 163 million years ago (Mya) (Fig. 1A), which is consistent with the long-term study of Lepidoptera chromosomes [10–14].

Since most of the extant chromosomes (chromosomal-scale assemblies) were named by size, we next sought to facilitate the exploration of chromosome evolution by renaming more extant chromosomes (except 67 W chromosomes) based on their homology with ancestral chromosomes determined in the previous step (Additional file 1: Table S3). To this end, if no inter-chromosomal rearrangement was detected between an ancestral chromosome and an orthologous extant chromosome, the ancestral chromosome name is used for this extant chromosome. Extant chromosomes formed by fusion events are represented by a lowercase letter F. In contrast, chromosomes formed by fission are represented by the lowercase letter D (Fig. 1). For example, an extant chromosome formed by the fusion of ancestral chromosomes 1 and 2 is named 1f2 (Fig. 1A). Two extant chromosomes formed by the fission of ancestral chromosome 3 are called 3d1 and 3d2 (Fig. 1B). An extant chromosome that maintained ancestral chromosome 4 without inter-chromosomal rearrangement is named 4 (Fig. 1C). Figure 1 also shows more complex rearrangements that occurred only in a limited subset of species (Fig. 1D).

Following this rule, we renamed more than 5000 extant chromosomes in Lepidoptera. This systematic nomenclature greatly facilitated the research of chromosomal evolution (Fig. 1). To establish a chronological order for the evolutionary processes identified in lepidopteran chromosomes, each fusion and fission event of 31 ancestral chromosomes was marked on the species tree (Fig. 1). Some inter-chromosomal rearrangements that occurred at family level ancestral nodes were shared by extant species in that family, such as Sphingidae, Lycaenidae, and Tortricidae. In contrast, most inter-chromosomal rearrangements are lineage-specific (Fig. 1 and Supplementary Table S3). Notably, Noctuidae species, except for the Brick moth (*Agrochola circellaris*), all retained the ancestral karyotype without evidence of chromosomal fusion or fission (Fig. 1 and Additional file 1: Table S3). In addition to the 91 species that retained the ancestral karyotype, 88 species had karyotypes shaped by fusion events, which accounted for the largest proportion of inter-chromosomal rearrangement in Lepidoptera (Fig. 1 and Additional file 1: Table S3). Six species (*Antheraea pernyi*, *Lysandra bellargus*, *Lysandra coridon, Leptidea sinapis, Philereme vetulata*, and *Tinea semifulvella*) distributed across five families were found to have chromosome numbers greater than 31 (Fig. 1 and Additional file 1: Table S3).

### Characteristics associated with chromosomal rearrangements

**Fig. 2:**
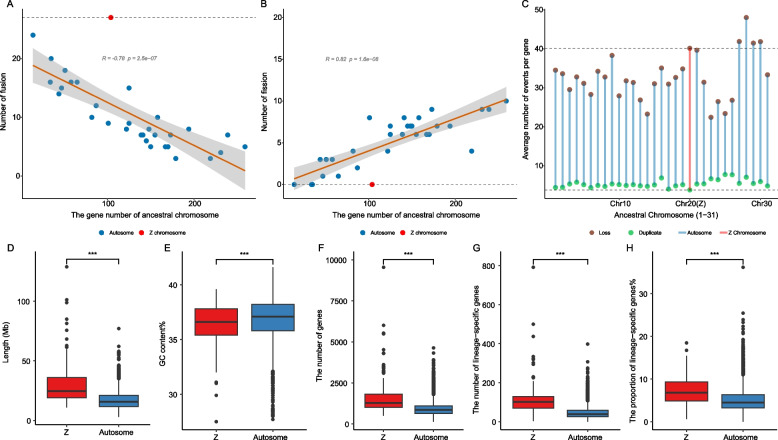
The distinctive patterns of the Z chromosome in rearrangement and gene evolution. **A** There are a total of 31 points, with each ancestral chromosome corresponding to a single point. The Z chromosome is marked with red, and the dotted line marks its position relative to the autosomes. The *x*-axis is the number of genes an ancestral chromosome contains, and the *y*-axis is the number of fusions that have occurred independently in the evolutionary history of the ancestral chromosome. There was a negative correlation (Pearson’s product-moment correlation, *P* < 0.001) between the number of fusion events and the gene number of ancestral chromosomes. **B** There are a total of 31 points, with each ancestral chromosome corresponding to a single point. The Z chromosome is marked with red, and the dotted line marks its position relative to the autosomes. The *x*-axis is the number of genes an ancestral chromosome contains, and the *y*-axis is the number of fissions that have occurred independently in the evolutionary history of the ancestral chromosome. There was a positive correlation (Pearson’s product-moment correlation, *P* < 0.001) between the number of fission events and the gene number of ancestral chromosomes. **C** There are a total of 62 points, with each ancestral chromosome corresponding to two points. The Z chromosome is marked with red, and the dotted line marks its position relative to the autosomes. The *x*-axis is 31 ancestral chromosomes, and the *y*-axis is the average number of loss or duplication events per gene of an ancestral chromosome. The gene loss events are marked in brown, and the gene duplication events are marked in green. **D** The boxplot shows that the length of extant Z chromosomes is significantly (Wilcoxon rank-sum test “greater”; *P* < 0.001) higher than that of the extant autosome. **E** The boxplot shows that the GC content of extant Z chromosomes is significantly (Wilcoxon rank-sum test “greater”; *P* < 0.001) higher than that on the extant autosome. **F** The boxplot shows that the number of genes of extant Z chromosomes is significantly (Wilcoxon rank-sum test “greater”; *P* < 0.001) higher than that on the extant autosome. **G** The boxplot shows that the number of lineage-specific genes on extant Z chromosomes is significantly (Wilcoxon rank-sum test “greater”; *P* < 0.001) higher than that on the extant autosome. **H** The boxplot shows that the proportion of lineage-specific genes of extant Z chromosomes is significantly (Wilcoxon rank-sum test “greater”; *P* < 0.001) higher than that on the extant autosome

We counted the number of fusion and fission events of each ancestral chromosome during the evolution of 185 lepidopteran species (Fig. 2A, B). This analysis showed that all 31 ancestral chromosomes had fusion events. We observed a significant negative correlation between the number of chromosome fusion events that occurred during the evolution of an ancestral chromosome and the number of genes it contains (Pearson’s product-moment correlation, *P* < 0.001; Fig. 2A). Conversely, we found a significant positive correlation between the number of chromosome fission events and the number of genes in the ancestral chromosome (Pearson’s product-moment correlation, *P* < 0.001; Fig. 2B). Based on the fact that GC pairs have one additional hydrogen bond compared to AT pairs, it is expected that chromosomes with high GC content would exhibit increased stability [63, 64] and experience fewer instances of fission [12]. To test this hypothesis, we investigated whether chromosome size was correlated with GC content, and found a statistically significant negative correlation between chromosome length and GC content in most species studied (155 out of 185 species; Pearson’s product-moment correlation,* P* < 0.05; see Additional file 1: Tables S1 and S3). This suggests that the occurrence of chromosome fission is inversely related to GC content.

**Fig. 3:**
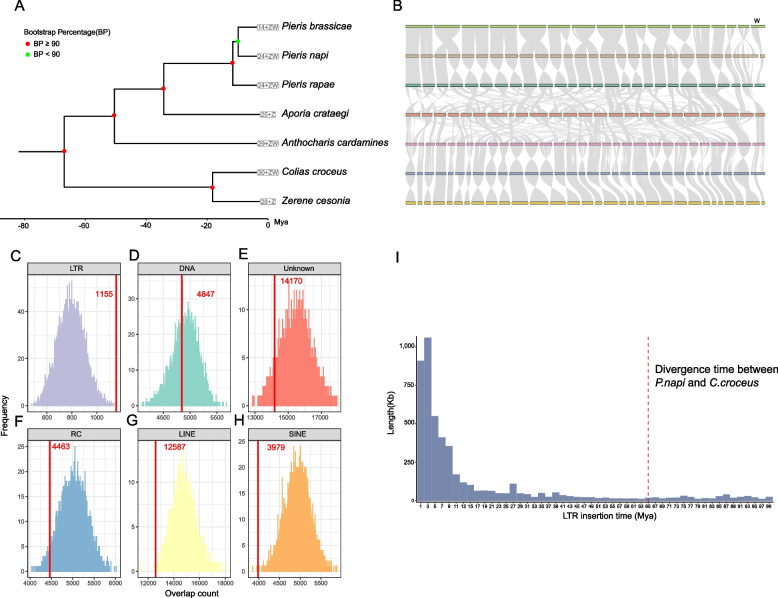
Chromosome rearrangement is associated with LTR insertion. **A** The species tree of *Pieridae*. **B** The synteny plots of *Pieridae*. We found that some species of *Pieridae* experienced extensive chromosomal rearrangements, such as *P. napi, Pieris brassicae, Pieris rapae*, and *Aporia crataegi*. Meanwhile, the synteny within the genus *Pieris* is highly conserved. **C** LTRs are enriched within *C. croceus* and *P. napi* synteny breakpoint regions in the *P. napi* genome. Histograms show the distribution of TE counts (by class) in 10,000 randomized sets of chromosomal regions with the same size distribution as observed in *C. croceus* and *P. napi* synteny breakpoint regions. Red lines indicate observed values for each TE class within autosomal synteny breakpoint regions, which shows that long terminal repeat retrotransposons (**C** LTR) are significantly enriched in the breakpoint regions. The DNA transposons (**D** DNA), unidentified transposons (**E** Unknown), rolling-circle Helitron transposons (**F** RC), and long and short interspersed nuclear elements (**G** LINE and **H** SINE) are not enriched. **I** The time of the burst of LTRs on *P. napi* genome is later than the divergence time between *P. napi* and *C. croceus.* Histograms showing the age distribution of LTRs located on the *P. napi* genome. Red lines indicate the divergence time between *P. napi* and *C. croceus*. The *y*-axis shows the LTR content (kb) of the *P. napi* genome

In addition, we found that some species of Pieridae, such as *P. napi*, *Pieris brassicae*, *Pieris rapae*, and *Aporia crataegi*, did not share the relatively conserved chromosomal synteny observed in other Lepidoptera (Fig. 3A, B). These species have undergone highly complex chromosome rearrangements to the extent that, based on the available data, we cannot disentangle the fusion and fission events that have occurred in their chromosomes during evolution. Although previous research on gene content in *P. napi* showed extensive rearrangement in large synteny blocks [15], in the current study, we observed a high degree of conservation in chromosome synteny within the genus *Pieris* (Fig. 3B). In contrast, the chromosomes of *A. crataegi* underwent different extensive rearrangements (Fig. 3B). Since the loss of genes that contribute to DNA repair function may lead to extensive rearrangements [23], we examined DNA repair-related genes. We found no significant difference in the number of these genes between species with and without chromosomal rearrangement (Additional file 1: Table S4).

Repetitive elements can promote chromosomal rearrangement through nonallelic homologous recombination [18–22]. To investigate whether TEs contribute to lepidopteran evolution, we compared synteny between the genomes of *P. napi* and *Colias croceus,* the only member of its family that has retained the ancestral chromosome karyotype, to identify synteny breakpoint regions (hereafter referred to as breakpoint regions) in *P. napi*. These breakpoint regions (excluding chromosome ends) accounted for 22 Mb (6.9%) of chromosome sequence in *P. napi,* with an average length of 147 kb (*n* = 150, min = 3.7 kb, max = 2.4 Mb). Examination of six types of TEs’ distribution in the *P. napi* genome revealed that long terminal repeat (LTR) retrotransposons were highly enriched (permutation test, *P* < 0.001) in breakpoint regions compared to the expectation of random occurrence in the *P. napi* genome (Fig. 3C). Among all types of transposons, only LTRs were enriched at breakpoint regions, while unidentified transposons (Unknown), rolling-circle Helitron transposons (RC), and long and short interspersed nuclear elements (LINE and SINE, respectively) were not enriched (Fig. 3D–H). In addition, the large majority (more than 80%) of LTRs in the *P. napi* genome were inserted after it diverged from *C. croceus* (Fig. 3I).

Further exploration of TE distribution at breakpoint regions between *C. croceus* and the other three Pieridae species (*P. brassicae, P. rapae*, and *A. crataegi*) showed that LTRs were consistently significantly enriched (permutation test, *P* < 0.05) in these breakpoint regions except for in *P. brassicae* (permutation test, *P* < 0.0785), while the distribution of DNA, Unknown TEs, SINE, LINE, and RC elements was not significantly enriched (Additional file 2: Fig. S2). Moreover, the insertion of the large majority of LTRs also occurred after the speciation of these three taxa (Additional file 2: Fig. S2). Taken together, the above evidence suggests that the recent proliferation of LTRs in these species’ genomes may be related to the extensive chromosomal rearrangements in these species.

### The distinctive patterns of the Z chromosome in rearrangement and gene evolution

Our investigation into inter-chromosomal rearrangements during the evolution of 185 species of Lepidoptera revealed 24 independent fusion events of the ancestral chromosome 20, the Z chromosome (Fig. 2A, B). However, we did not observe any instances of its fission. We conducted further investigations into the differences between Z chromosomes and autosomes and found that the length of the Z chromosomes in extant species is significantly larger than that of autosomes (Wilcoxon rank-sum test “greater,” *P* < 0.001; Fig. 2D, Additional file 1: Table S3), while the GC content is significantly lower than that of autosomes (Wilcoxon rank-sum test “less,” *P* < 0.001; Fig. 2E, Additional file 1: Table S3). Based on the above findings, chromosomes with longer lengths and lower GC content should undergo more chromosome fission and fewer chromosome fusion events during evolution. However, the rearrangement patterns of the Z chromosome exhibit distinct characteristics.

In the ancestral chromosomes of Lepidoptera, the number of genes on the Z chromosome ranked only 20th (Figs. 1 and 2C). This implies that the extant Z chromosome harbors a smaller number of highly conserved genes (i.e., those derived from ancestral homologs) than the autosomes. This may indicate that highly conserved genes on the Z chromosome are lost more rapidly during evolution than highly conserved genes on autosomes. We conducted an extensive analysis focusing on the occurrences of gene loss and duplication events across ancestral genes situated on each ancestral chromosome throughout evolution. Specifically, the absence of a homologous gene in the present-day species designates the loss of an ancestral gene during the evolution of said species. Conversely, the presence of two homologous copies of an ancestral gene in a present-day species (limited to cases where ancestral genomes were reconstructed using orthogroups featuring two or fewer gene copies) indicates a duplication event during the evolutionary history of that species. Our investigation, spanning 185 species genomes, reveals noteworthy patterns. Genes located on the ancestral Z chromosome exhibit a higher frequency of loss events, averaging 40.15 loss events per gene. This positions the ancestral Z chromosome as the fifth highest in loss events among the set of 31 ancestral chromosomes (Fig. 2C). In contrast, the ancestral Z chromosome showcases the lowest incidence of gene duplication events, ranking last among the 31 ancestral chromosomes with an average of 3.83 gene duplication events per gene (Fig. 2C). The unique evolutionary pattern may account for the lower number of highly conserved genes on the extant Z chromosome relative to autosomes.

Conversely, the number of genes present on the extant Z chromosome is significantly greater than that on autosomes (Wilcoxon rank-sum test “greater,” *P* < 0.001; Fig. 2F, Additional file 1: Table S3). Building upon the previous analysis, we postulate that this may be attributed to the faster accumulation of less-conserved genes, such as lineage-specific genes, on the Z chromosome compared to autosomes. We further analyzed the number of lineage-specific genes (i.e., genes unique to one species and likely represent new genes relative to ancestral genes, with no homolog detected among the other 186 selected species) on each extant chromosome. We calculated the proportion of lineage-specific genes relative to all genes on the respective chromosome. Our results indicate that the number and proportion of lineage-specific genes on the extant Z chromosome are significantly higher than those on the autosomes (Wilcoxon rank-sum test “greater,” *P* < 0.001; Fig. 2G, H, Additional file 1: Table S3), providing evidence that the Z chromosome can accumulate lineage-specific genes more rapidly during evolution. This may explain why the extant Z chromosome contains more genes than the autosomes. These cumulative results revealed the distinctive evolutionary patterns of the Z chromosome.

### Common origin of W chromosomes and formation of neo-W chromosome

**Fig. 4:**
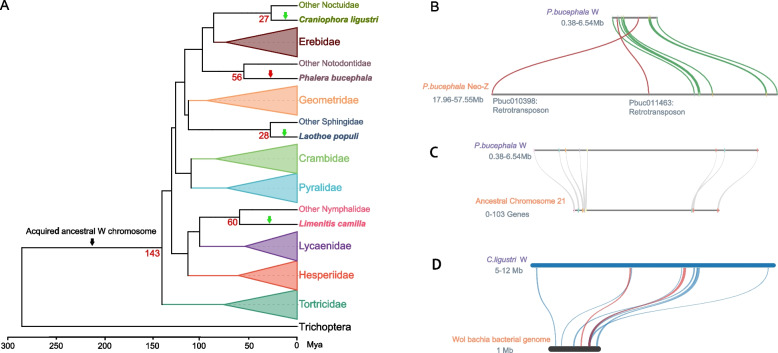
Origin of W chromosomes and formation of neo-W chromosome. **A** The simplified Phylogenetic Tree of Lepidoptera with Latin names painted in different colors represents species of different families. The black arrow represents Lepidoptera acquired ancestor W chromosome 143 million years ago. The red arrow represents *P. bucephala* formed neo-W chromosome through chromosome fusion with ancestral chromosome 21 within 56 million years. The green arrows represent the W chromosomes acquired in the *Wolbachia* bacterial genome through horizontal transfer. *C. ligustri* acquired the *Wolbachia* bacterial genome within 27 million years, *L. populi* acquired *Wolbachia* bacterial genome within 28 million years, and *L. camilla* acquired *Wolbachia* bacterial genome within 60 million years. **B** The 11 orthologs between *P. bucephala* W chromosome and *P. bucephala* Neo-Z chromosome. The orthologous relationships of two retrotransposons were marked with red. **C** The eight orthologs between *P. bucephala* W chromosome and ancestral chromosome 21. **D** Blue and red links indicate synteny blocks of the *Wolbachia* bacterial genome and the W chromosome of *C. ligustri,* in forward and reverse orientation, respectively

Among the 185 Lepidoptera genomes examined in this study, complete sequence assemblies of the W chromosome were available for 67 species, spanning 16 families. It is worth noting that the absence of the W chromosome in the current assemblies is not an indicator of the W chromosome being absent in the species. Previous studies have often relied on the dissimilarity of W chromosome sequences between two species as evidence supporting the independent origins of their respective W chromosomes [41]. However, it is also possible for two W chromosomes derived from the same ancestral source to have evolved into entirely different sequences over time. We conducted all vs. all collinearity analysis of protein-coding genes among 67 W chromosomes. Our findings reveal the presence of collinear blocks among W chromosomes in most species, while others lack such blocks, indicating the absence of segments shared by all W chromosomes (Additional file 1: Table S6). However, we have still found evidence that suggests the origin of these W chromosomes can be traced back to a common ancestral W chromosome. For example, the W chromosome of *Laothoe populi* was relatively long (19.4 Mb) and shared at least one collinear block (contained at least 7 collinear genes) with the W chromosomes of 64 other species. In contrast, the W chromosomes in the remaining two species (*Dryobotodes eremita* and *Marasmarcha lunaedactyla*) had at least one collinear block shared with at least one of the 63 other species (for instance, these two share 39 and 14 collinear blocks with the W chromosome of *Eupsilia transversa*). The average number of collinear blocks between 67 W chromosomes is 31.8, and the average number of collinear genes is 287.3 (Additional file 1: Table S6). Thus, combining phylogenetic and synteny analyses, we speculated that these W chromosomes of Ditrysia originated from the same ancestral W chromosome at least 143 Mya, with different sequences lost during subsequent evolution, ultimately resulting in the absence of any single fragment shared among all extant W chromosomes (Fig. 4A and Additional file 1: Table S6).

Since Lepidoptera ancestors did not have W chromosomes, most of the 67 analyzed W chromosomes do not have any orthologs with the reconstructed ancestral genome. However, Buff-tip (*Phalera bucephala*) has eight genes orthologous to those on the reconstructed ancestral chromosome 21, more than that in other species. These orthologs were distributed in the head and tail of the chromosome (Fig. 4C), and based on the above criteria, the W chromosome was therefore considered orthologous to ancestral chromosome 21. Buff-tip also harbors a neo-Z chromosome derived from a fusion of ancestral chromosome 21 and the ancestral Z chromosome (Fig. 1, Additional file 1: Table S2). Thus, the W chromosome of Buff-tip also has at least 11 orthologous genes with its neo-Z chromosome, which, except for two transposons, are sequentially distributed in the head and tail of the homologous autosomal region (Fig. 4B). This also means that this new part of the W chromosome has experienced massive gene loss. Among the 11 genes mentioned above, the W chromosome also carried Pbuc008584, a gene of unknown function orthologous to genes on autosome 21 of 114 species, but without an orthologous gene on the Buff-tip neo-Z chromosome (Additional file 1: Table S2). This finding suggested that the orthologous gene on the neo-Z chromosome was lost, which is now a female-specific gene in Buff-tip. These results illustrated that chromosomal fusions lead to the formation of neo-sex chromosomes in Buff-tip. Thereafter, we compared the substitution rates between the Buff-tip Z and W gametolog and the corresponding lesser swallow prominent (*Pheosia gnoma*) ortholog (i.e., Z to the lesser swallow prominent vs. W to the lesser swallow prominent). The W gametologs showed higher Ka/Ks to the lesser swallow prominent than did Z gametologs (Wilcoxon rank-sum test “greater,” *P* < 0.05), which are in line with purifying selection being less efficient on the W than on the Z chromosome (Additional file 1: Table S12).

### Horizontally transferred genes from Wolbachia to W chromosomes

In the 67 W chromosomes of 67 species, annotation with eggNOG-mapper [65] identified 40,934 genes with annotation information, while 45,307 lacked annotation information. Among the 40,934 annotated genes, 97.6% were eukaryotic, while 2.4% appeared to be derived from bacteria. Three species had more than ten putative bacterial genes on their respective W chromosomes, with *Limenitis camilla* notably carrying 84 genes from *Wolbachia*, 35 of which had introns; the W chromosome of *Craniophora ligustri* had 133 genes from *Wolbachia*, 43 of which had introns; and the W chromosome of *Laothoe populi* had 38 genes from *Wolbachia*, of which eight had introns (Additional file 1: Table S7). Since *Wolbachia* genomes usually do not contain introns, meaning that these introns were likely obtained after integrating into the insect genomes. HGT-acquired genes containing introns demonstrate significantly higher expression levels than genes without introns [5], suggesting that these intron-containing genes may have played critical roles. Additionally, we did not identify any *Wolbachia* genes shared by all three W chromosomes. Still, we did reveal a limited subset of genes shared by two species that generally encoded structural proteins of *Wolbachia*. More specifically, the W chromosomes of both *L. camilla* and *C. ligustri* had ABC transporter genes from *Wolbachia*, including five in *L. camilla* and two in *C. ligustri,* all of which contained introns except for one in *C. ligustri* and may be involved in adaptation to environmental challenges (e.g., detoxification). The W chromosome of *L. populi* had two DNA methyltransferase genes from *Wolbachia* that all contained introns. *L. populi* has four DNA methyltransferase genes, while most species (155 out of 185 species) have no more than two. *Wolbachia* has feminizing or male-killing functions in many insects (including in Lepidoptera) [66–73], and the W chromosome is sex-determining in many species of Lepidoptera. We speculated that these W-linked DNA methyltransferase genes from *Wolbachia* may play an essential role in sex determination [69, 74]. Verification by NCBI BLASTP with Non-Redundant Protein Database [54] indicated that at least five of the top ten alignment results were consistent with our annotation (Additional file 1: Table S7).

Subsequently, we retrieved the *Wolbachia* endosymbiont genome of moths from NCBI, specifically the entry labeled GCF_018141665.1, which originates from *Spodoptera*. Unfortunately, the *Wolbachia* genomes for the aforementioned three species were not available. Therefore, we employed this particular genome as an approximate replacement for analysis purposes. To assess the collinearity between the *Wolbachia* genome and the three W chromosomes, we utilized MCScanX (Fig. 4D, Additional file 2: Fig. S3). There were several collinear blocks between each of the three W chromosomes and the bacteria genome, indicating that these genes do not undergo extensive reshuffling after being inserted into the W chromosome. These ostensibly inserted sequences were centrally distributed in several W chromosome regions of *C. ligustri* and centrally distributed in one region of W chromosomes of *L. populi* and *L. camilla* (Fig. 4D, Additional file 2: Fig. S3). These findings suggested that specific sequences located on the W chromosome of *C. ligustri*, *L. populi*, and *L. camilla* may have been acquired through horizontal gene transfer from the genome of the endosymbiotic bacteria *Wolbachia*. This event facilitated the emergence of neo-W chromosomes in these organisms, which may have impacted their ability to adapt to the environment as well as their sex-determination mechanisms.

### The gene movements in Lepidoptera

**Fig. 5:**
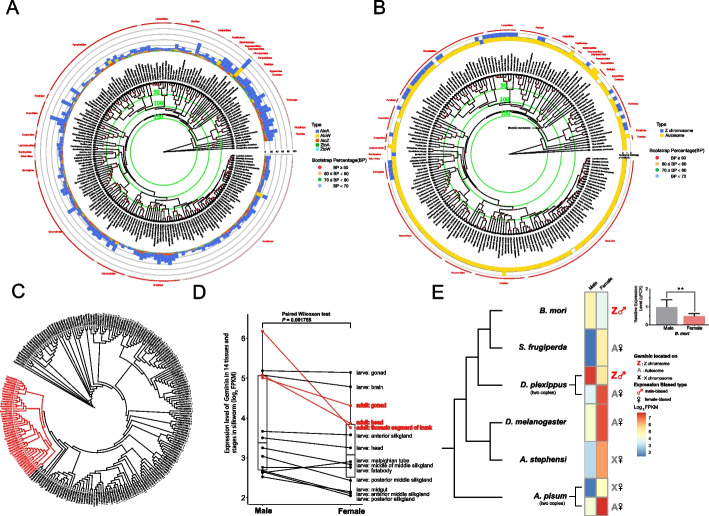
The gene movements in Lepidoptera. **A** The species tree with the stacked bars indicates the number of gene movements of different types (between autosomes, autosomes to Z chromosomes, Z chromosomes to autosomes, autosomes to W chromosomes, and Z chromosomes to W chromosomes) across Lepidoptera. **B** The species tree with the stacked bars indicates the number of *Geminin* located in Z chromosomes and autosomes across Lepidoptera. The black arrow represents the *Geminin* movement event that occurred between 152.1 and 141.7 Mya. **C** The gene tree of *Geminin*. *Geminins* located on Z chromosomes are marked with red. **D** Comparison of gene expression of the *Geminin* in 14 tissues and stages in silkworm. The average FPKM of biological repeats is shown in the figures. Male-biased expression is more evident in adults, marked with red. **E** *Geminin* location and expression in Lepidoptera and other order insects. The Capital letters with different colors represent the chromosomes *Geminin* located, and the sexual logo with different colors represents the expression biased type. *Danaus plexippus* and *Acyrthosiphon pisum* had two *Geminin* copies in other chromosomes. The average FPKM of biological repeats is shown in the figures. The picture shows the transcriptome analysis and qPCR results of the head of the adult silkworm and the transcriptome analysis results of the gonads of the adult monarch butterfly and the whole body of the adult fall armyworm. More information on the developmental stage and tissue for the transcriptome data for each species are given in Additional file 1: Tables S9 and S10

In addition to large-scale chromosome rearrangement, the movement of individual genes between chromosomes also alters the relative positions of genes. Utilizing homology comparisons between ancestral and extant chromosomes, we identified a total of 3915 instances of inter-chromosomal gene movement events (Additional file 1: Table S8). The occurrence of gene movement events varied substantially across species (Fig. 5A). Within the 185 species under examination, the highest count of observed gene movement events (a total of 92) was recorded in *Marasmarcha lunaedactyla*, while *Autographa gamma* exhibited one of the lowest occurrences (a total of 4), all of which were autosomal. Moreover, a significant positive correlation (assessed through Pearson’s product-moment correlation, with a significance level of *P* < 0.001) was observed between the number of gene movement events and the gene count within each species (Additional file 1: Table S1). Most gene movements (a total of 3042) transpired between autosomes, followed by gene transfers from autosomes to Z chromosomes (a total of 582) and from autosomes to W chromosomes (a total of 163). Comparatively, fewer movements were associated with the transfer from Z chromosomes to autosomes (a total of 115) and from Z chromosomes to W chromosomes (a total of 13) (Fig. 5A and Additional file 1: Table S8). It is worth noting that our analysis focused on the movement events of the orthologous genes derived from ancestral genes within the ancestral reference genome for Lepidoptera. These genes were shared across a majority of extant lepidopteran species. However, the study excluded less conserved genes, including lineage-specific genes. Among the 3915 genes transferred between chromosomes, 2541 had identifiable parental genes, while the remaining 1374 lacked paralogs (Additional file 1: Table S8). Additionally, genes transferred from autosomes to the W chromosome, lacking identifiable parental genes on autosomes or Z chromosomes, were identified as female-specific genes. These particular genes might hold significant roles in shaping sexual dimorphism and contributing to sex determination.

Transpositions of genes between sex chromosomes and autosomes may have a higher impact on gene expression and function than transpositions between autosomes [43–51]. To assess this, we focused on the most frequent occurrence of gene movement between Z chromosomes and autosomes, the translocation of *Geminin* from an autosome 10 to the Z chromosome. The ancestral autosomal gene, *Geminin,* is situated on autosome 10 in 179 species, including two Trichoptera species while possessing Z chromosomal homologs in 42 species (Fig. 5B). To distinguish between the hypotheses that the observed *Geminin* movements occurred as a singular event at an ancestral node and were inherited by descendants, or that they arose independently multiple times at distinct nodes, we constructed a phylogeny of *Geminins*. The gene tree indicated that the *Geminins* were clustered into two categories based on the positions rather than the phylogenetic relationship between the species: the Z chromosome cluster and the autosome 10 cluster (Fig. 5C). This suggested that the first hypothesis was correct and that the transfer of *Geminin* from the autosome 10 to the Z chromosome took place only once at an ancestral node. Combining the species tree and gene tree, we suggest that this movement event occurred between 152.1 and 141.7 Mya (Fig. 5B). Following this *Geminin* duplication event, some lineage-specific *Geminin* loss or duplication events took place in various descendants (Fig. 5B).

In the silkworm genome, *Geminin* is encoded by Bmor011058 (InSectBase2.0 gene ID, corresponding to BMSK0000275 in Silkdb3.0 [75]), which is located in the region of 8,741,726–8,745,655 bp of the Z chromosome, and had no parental gene on autosome 10 (Fig. 5B, C). Previous reports showed that *Geminin* overexpression leads to weight loss in the silkworm, while its knockdown causes weight gain [76]. Since the male moths were smaller than the female moths [77], we examined *Geminin* expression in both sexes using 54 transcriptomes from 14 different tissues and developmental stages (Additional file 1: Table S9). The results revealed that males had significantly higher *Geminin* expression levels than females (Paired Wilcoxon test; *P* < 0.001, Fig. 5D, E), which was more pronounced in the adult stage. The smaller body size of adult male silkworms is consistent with the high expression of *Geminin* in males (Fig. 5D). Further analysis of transcription levels of *Geminin* in these three tissues of the adult silkworm by qRT-PCR confirmed this (*T*-test; *P* < 0.01) (Additional file 2: Fig. S4C). The above results indicate that the sex-biased expression of *Geminin* may be one of the factors contributing to the sexual dimorphism in the body size of adult silkworms.

Next, we asked whether this gene movement influences gene expression. We examined the expression level of *Geminin* in other insects. In the fall armyworm, *Spodoptera frugiperda*, only one copy of *Geminin* is found, encoding by Sfru017961 on autosome 10. Different from the high expression of *Geminin* in the male silkworm, transcriptome analysis showed that the expression of Sfru017961 in female moths is significantly higher than that in male moths (*T*-test; *P* < 0.001, Fig. 5E, Additional file 2: Fig. S4B, and Additional file 1: Table S9).

In another case, the monarch butterfly *Danaus plexippus* has two copies of *Geminin* with 65% sequence similarities (BLAST positives), one on the autosome 10 and the other on the Z chromosome. The autosome ortholog of *Geminin* is encoded by LOC116770314 on autosome 10, and the Z chromosome paralog of *Geminin* is encoded by LOC116777402 located in the region of 7,693,468–7,695,486 bp of Z chromosome. At the downstream of LOC116777402, we found a retrotransposon (LINE/Penelope) situated in the region of 7,696,590–7,696,659 bp of Z chromosome. Possibly, the *Geminin* paralog was copied from chromosome 10 and then inserted into the Z chromosome with the assistance of a retrotransposon. Interestingly, we found that LOC116770314, the orthologous copy on the autosome, shows significant female-biased expression (*T*-test; *P* < 0.05), whereas LOC116777402, the paralogous copy on the Z chromosome, shows significant male-biased expression (*T*-test; *P* < 0.01) in the gonads of adults (Fig. 5E, Additional file 2: Fig. S4A and Additional file 1: Table S9).

Further exploration showed that *Geminin* was located on the X chromosome in mosquitoes and aphids, and this gene shows female-biased expression in *Drosophila melanogaster*, *Anopheles stephensi*, and *Acyrthosiphon pisum,* which were male heterogamety (XY or XO for male) (Fig. 5E, Additional file 1: Table S10). These results indicate that *Geminin* displays a prevalent sex-biased expression across various insect species. However, this sex-biased expression of *Geminin* is affected by the gene’s chromosomal location and, as a result, may contribute to divergent sexual dimorphism across different insect lineages.

## Discussion

Previous studies pertaining to chromosomal evolution in Lepidoptera have relied on a limited number of species or even a single species as a reference [12, 15, 64]. This results in a bias toward the selected reference species. In this study, we reconstructed an ancestral reference genome for Lepidoptera, facilitating the study of evolutionary processes of lepidopteran chromosome evolution independent of any single extant species. However, with an increased number of species, it is challenging to reconstruct ancestral chromosomes. This is because of the decreased availability of highly conserved genes. Consequently, a reduced number of ancestral genes are available to be incorporated into the reconstructed ancestral chromosomes. While our reconstructed ancestral genome had fewer genes than the actual ancestral genome, this reconstructed genome and the threshold used for orthologous chromosome detection (those sharing at least eight orthologous genes with an ancestral chromosome) are sufficient to support our findings on macro-evolutionary patterns of the genome of Lepidoptera, highlighted by our collinearity analysis. We produced a synteny analysis comparing all 187 species to validate the correctness of chromosomal orthologous relationships detected through our reconstructed ancestral genome. While we selected a large number of high-quality genomes considering the diversity of Lepidoptera species, data analysis bias may be present due to the uneven distribution of species across some lineages.

Extant chromosomes are usually numbered according to their descending length in genome assemblies [78–82]. However, this nomenclature lacks evolutionary information and is thus sub-optimal for large-scale chromosome evolution studies. To ensure that orthologous chromosomes were more readily identifiable across species regardless of changes in their length during the evolution of extant species, in the present study, chromosomes in extant species were renamed based on their homology with ancestral reference chromosomes (Additional file 1: Table S3), incorporating likely fusion and fission events. This ancestral chromosome reconstruction combined with designations for extant chromosomes according to homology with the ancestral chromosomes is suitable to be extended to a broader range of animal groups with deeply conserved chromosomal synteny, such as birds, reptiles, and mammals [23, 62, 83–86]. Moreover, this nomenclature improves the convenience and coherence of chromosome evolution research and representation, potentially accommodating tens of thousands or millions of chromosomes in a single study.

Mounting evidence supports the idea that sex chromosomes differentiate faster than autosomes [24–29, 31]. Previous studies comparing orthologous genes have outlined a “faster-X/Z effect” during which loci on sex chromosomes evolve faster than comparable loci on autosomes, or a “large-X/Z effect,” during which sex chromosomes are disproportionately involved in reproductive isolation as well as adaptation [24–29, 31]. Our research has allowed for additional insights into the distinct evolutionary characteristics of the Z chromosome in comparison to autosomes. Specifically, a higher frequency of fusion events and a lower frequency of fission events in the evolution of the Z chromosome were observed. Furthermore, our research revealed that the Z chromosome of Lepidoptera underwent a more rapid loss of conserved genes, an increased acquisition rate of lineage-specific genes, and a slower pace of gene duplication compared to autosomes. These specific evolutionary patterns are most likely due to various factors, including dosage compensation, accelerated molecular evolutionary rates, and the inhibition of chromosome recombination [24–33, 87].

Since the W chromosome typically harbors many transposons, lacks functional genes, and is enriched with repeat sequences, its origin and evolution remain largely unknown [34–38]. Our synteny analysis of 67 W chromosomes suggested that these W chromosomes may have originated from the same ancestor W chromosome. Previous studies indicated that the W chromosomes in the Tischeriidae and Ditrysia lineages had independent origins [41], consistent with our findings, as our study only involved Ditrysia. However, our results also suggest that as more W chromosome sequences from Tischeriidae are made publicly available, comparative genomic analysis of the W chromosomes in both the Tischeriidae and Ditrysia lineages on a larger scale may reveal evidence of a common origin for the W chromosomes in both lineages. Characterization of four neo-W chromosomes revealed that one in *P. bucephala* was potentially generated through sex chromosome-autosome fusion. At the same time, the other three may acquire the *Wolbachia* bacterial genome by horizontal transfer. Several pieces of evidence confirmed that these *Wolbachia* sequences integrated into W chromosomes rather than contamination. First, the third-generation sequencing platform PacBio assembled these W chromosomes, which offered long-length reads. Second, Hi-C analysis located the *Wolbachia* sequences to the W chromosome. This excluded the possibility of bacterial contamination. Third, these *Wolbachia*-originated genes contain introns. We found that inserted sequences mapped to the *Wolbachia* genome had genes encoding ABC transporters and DNA methyltransferases. These ABC transporters on the W chromosome could potentially increase female environmental toxin tolerance. DNA methyltransferases play a crucial role in epigenetic regulation, which means that the inserted bacterial genome may have a significant impact on the gene expression of this species [74]. We speculate that insertion of the *Wolbachia* genes into W chromosomes might enhance female-specific toxin tolerance, as well as epigenetic regulatory effects promoting feminine traits. Our results provide new insights into the complex evolutionary dynamics of sex chromosomes in the animal kingdom.

Previous studies indicated more gene movement events from sex chromosomes to autosomes, such as research regarding retro-genes moved out of the Z chromosome in the silkworm [49, 52]. Here, we show that the characteristics of gene movement events vary substantially across species. Our analyses revealed the movements of orthologs of ancestral genes across species, but the actions of lineage-specific genes were not investigated in our study. This differs from previous studies focusing on retro-genes’ movement, including many lineage-specific genes [49, 52]. Additionally, we identified a list of genes transferred from the autosome to the W chromosome and part of them without parental genes on the autosome, resulting in these genes becoming female-specific. Further research is needed to explore the function of these genes, and this gene list can guide future study of sexual dimorphism and sex determination in lepidopteran insects.

## Conclusions

We reconstructed a lepidopteran ancestral reference genome and introduced a novel chromosome nomenclature. This allowed us to rename more than 5000 extant chromosomes, unveiling the historical events of chromosomal rearrangements and their characteristics within Lepidoptera. Our research revealed that, in comparison to autosomes, the Z chromosome in Lepidoptera experienced rapid loss of conserved genes, quick acquisition of lineage-specific genes, and a low rate of gene duplication. Furthermore, we provided evidence suggesting that all 67 available W chromosomes share a common ancestral chromosome, with four neo-W chromosomes identified. We also identified approximately 4000 instances of inter-chromosomal gene movement. Notably, the gene *Geminin* transferred from an autosome to the Z chromosome, showing female-biased expression on the autosome and male-biased expression on the Z chromosome, contributing to the sexual dimorphism of body size in silkworms. Overall, our study sheds light on the complex evolutionary history of lepidopteran chromosomes through ancestral chromosome reconstruction and a novel chromosome nomenclature.

## Methods

### Data collection and gene annotation

A total of 185 Lepidoptera and two Trichoptera genome assemblies at the chromosome-level were obtained from InsectBase 2.0 [5] and NCBI [54]. These assemblies had scaffold N50 > 5 Mb with sex chromosomes labeled. Of these, 92 genomes were obtained with known annotated official gene sets. The other 95 genomes were annotated by the BRAKER2 [55] pipeline C using both de novo and homology-based evidence using parameters “braker.pl –species = species1 –genome = genome.fasta –prot_seq = proteins.fasta –softmasking –gff3 –cores = 30”. For repeat sequence annotation, we first constructed lineage-specific de novo repeat libraries using RepeatModeler v2.0.2a using the LTR structural discovery pipeline [88]. We then identified and masked repeat sequences across genomes using RepeatMasker v4.1.2 [89] against the de novo lineage-specific repeat library generated by RepeatModeler2 and the RepBase v26.03 library [90]. We used eggNOG-mapper v2 [65] to perform functional annotation and clusterProfiler 4.0 [91] to perform enrichment analysis.

### Phylogenetic tree construction and divergence time estimation

We constructed a phylogeny of Lepidoptera using protein sequences from 187 assemblies. Only the longest transcript of each gene was retained in our analysis. OrthoFinder v2.3.14 [56] was used with Diamond v0.8.25 [57] to cluster proteins into orthogroups. MAFFT v7.475 [92] and IQTREE v2.2.0 [93] were then employed to estimate the species tree with Trichoptera as outgroups from 507 orthogroups and a minimum of 86.8% of species having single-copy genes in any orthogroup. R8s v1.81 [94] was used to estimate divergence time with the constrained divergence time range following TimeTree [95]. The construction method of the gene tree is the same as above. These results were visualized using GGTREE [96], ggplot2 [97], and iTOL [65].

### Synteny and substitution rates analysis

MCScanX v1.1 [98] was used for all vs. all synteny analysis between 187 species. For each comparison, we carried out a BLAST search of annotated protein sequences using DIAMOND v2.2.22 [57] with default parameters and ran MCScanX [98] with the parameters “-s10 -b 2.” To obtain the synteny relationship among W sequences, MCScanX with default parameters was used. These results were visualized with MCScan software [99] (https://github.com/tanghaibao/jcvi/wiki/MCscan-(Python-version)), SynVisio (https://synvisio.github.io/#/), and syntenyPlotteR [100]. We calculated the Ka/Ks between each of the Buff-tip Z and W gametolog and the corresponding lesser swallow prominent ortholog using KaKs_Calculator 3.0 [101] with the parameters “KaKs -i test.axt -o test.axt.kaks.”.

### Lepidoptera ancestral chromosome reconstruction and detection of orthologous chromosomes

Orthogroups with an excessive number of copies will interfere with the reconstruction of reference ancestral genomes [58]. Therefore, we selected orthogroups obtained from the above methods with no more than 2 copies in any of 187 species to reconstruct the lepidopteran ancestral chromosomes by using AGORA [58] with parameters “agora-generic.py species-tree.nwk orthologyGroups/*orthologyGroups.list genes/*genes.list”. According to previous studies and our synteny analysis results, the species’ chromosomes with karyotype 31 have not undergone an inter-rearrangement event. Next, we manually reorganized continuous ancestral regions (CARs) to 31 ancestral chromosomes based on gene synteny among CARs and species with karyotype 31 inferred using MCScanX_h [78, 98].

If an extant chromosome and an ancestral chromosome shared at least eight orthologous genes, we determined that they were orthologous chromosomes. Based on orthologous genes, we found the orthologous ancestral chromosomes for all extant chromosomes. Orthologous genes between extant genomes and the reference ancestral genome were compared using AGORA’s src/misc.compareGenomes.py script in “printOrthologuesList” mode. Karyotype plots were visualized with AGORA’s src/misc.compareGenomes.py script in “drawKaryotype” mode and syntenyPlotteR [100]. Groups of at least eight genes relocating to more than one chromosome in a descendant genome, and inversely, groups of at least eight genes from two or more ancestral chromosomes relocating on the same descendant chromosome were considered inter-chromosomal rearrangements. Because we were unable to sort out the rearrangement of some species (*A. crataegi, Aricia agestis, Brenthis ino, Hesperia comma, L. sinapis, Melinaea marsaeus, Melinaea menophilus, Operophtera brumata, P. brassicae, P. napi,* and *P. rapae*), those species were excluded when counting the number of inter-chromosome rearrangements.

If the ancestral chromosome corresponding to a gene was inconsistent with the ancestral chromosome corresponding to the orthologous extant chromosome where the gene is located, we identified that this gene had moved between the two chromosomes (Additional file 1: Table S8). For example, if gene *A* is located on chromosome 2, but its ancestral ortholog is located on ancestral chromosome 1, gene *A* was considered to move from chromosome 1 to chromosome 2 during evolution. If there is also an orthologous gene *B* located on chromosome 1 (which means gene *B* is the ancestral ortholog), gene *B* was considered to be the parental gene of gene *A.* Gene *A* may arise through a copy and paste mechanism (RNA-mediated (retrotransposed)). Otherwise, gene *A* may occur through a cut-and-paste mechanism (DNA-mediated) without a parental gene detected.

### Gene expression analysis

Transcriptome data for three species (silkworm, fall armyworm, and monarch butterfly) were collected from NCBI (Additional file 1: Table S9).

RNA-Seq reads were first filtered using Trimmomatic v0.38 [102]. The clean reads were then mapped to the genome using Bowtie2 v2.3.4 [103], and RSEM v1.3.0 [104] was used to calculate FPKM (fragments per kilobase of transcript per million fragments mapped). The average FPKM of biological repeats is shown (Fig. 5D and Additional file 2: Fig. S4A and B). These results were visualized with ggplot2 [97].

The *Geminin* expression data and location in other order insects were collected from InSexBase (Insect Sex Chromosome and Sex-Biased Genes Database) [105] by searching “geminin” in the gene search function.

### Silkworm strain

The Dazao silkworm strain was maintained at the Gene Resource Library of Domesticated Silkworm of Southwest University (Chongqing, China). They were housed with fresh mulberry leaves at 25 °C.

### Quantitative real time-PCR (qRT-PCR) assay

Total RNA was purified from silkworm tissues using an RNA Isolation Kit V2 (Vazyme, Nanjing, China) and reverse transcribed into cDNA using HiScript III RT SuperMix for qPCR (Vazyme, Nanjing, China). Primers corresponding to *Geminin* were used to investigate the transcription levels (Additional file 1: Table S11). The ribosomal protein gene (*rps18*) was used as an internal gene. The qPCR was performed in a 20μL reaction mixture containing 1μL of cDNA, 0.4 mM of each primer, and 2 × ChamQ Universal SYBR qPCR Master Mix (Vazyme, Nanjing, China) in each well of a 96-well plate. Three replicates of each test were performed.

The reaction conditions were 95 °C for 3 min, followed by 40 cycles at 95 °C for 15 s, 58 °C for 15 s, and 72 °C for 30 s. Finally, a melt curve was analyzed from 65 to 95 °C at 0.5 °C increments of 5 s each.

### Analysis of characteristics in breakpoint regions

To investigate the characteristics in breakpoint regions between *C. croceus* (the only species with ancestral karyotype in Pieridae) and *P. napi*, the TE content of synteny blocks and breakpoint regions between two species in *P. napi*’s genome were first investigated using BEDTools v2.28.0 [106] and the TE annotations described above. We defined the gaps between synteny blocks as breakpoint regions, excluded the chromosome ends that could have biased our analysis, identified TEs that overlap with the synteny blocks and breakpoint regions using BEDTools, and recorded TEs counts (independent summation) by categories for DNA, LINE, LTR, Rolling Loop, SINE, and Unknowns.

To examine the enrichment status of TEs within the breakpoint region, we simulated a set of random regions with the same size distribution as the observed breakpoint regions. We recorded their TE counts (10,000 replications). The *p*-value of each TE class was calculated by dividing the number of simulated sets in which TEs are equal to or greater than that of the same class in the observed breakpoint regions by the number of simulations (*n* = 10,000). We used the parseRM (https://github.com/4ureliek/Parsing-RepeatMasker-Outputs) program [107] to estimate the timing of LTR insertions. Moreover, we explored the distribution of TEs in breakpoint regions between *C. croceus* and the other three species (*P. brassicae*, *P. rapae*, and *A. crataegi*).

### Supplementary Information

**Additional file 1: Table S1.** The source of genome assemblies. **Table S2.** The information of ancestral genes. **Table S3.** The information of chromosome renaming. **Table S4.** The information of DNA repair related genes. **Table S5.** The information of species with karyotype of 31. **Table S6.** The information of all vs all synteny analysis of W chromosomes. **Table S7.** The information of W chromosome genes from Wolbachia bacterial genome. **Table S8.** The information of gene movements. **Table S9.** The information of *Geminin* expression and location in Lepidoptera. **Table S10.** The information of *Geminin* expression and location in other order insects. **Table S11.** The information of Primers. **Table S12.** The detailed data of Ka/Ks of gametologs.

**Additional file 2: Fig. S1.** The heat map of collinear genes. The number of collinear genes between any two species detected by MCScanX. **Fig. S2.** Chromosome rearrangement is associated with LTR insertion. Similar to Fig. 3. Histograms show the distribution of TE counts (by class) in 10,000 randomized sets of chromosomal regions. Red lines indicate real observed values for each TE class within autosomal synteny breakpoint regions which shows that long terminal repeat retrotransposons (LTR) are significantly enriched in the breakpoint regions. The DNA transposons (DNA), unidentified transposons (Unknown), rolling-circle Helitron transposons (RC), and long and short interspersed nuclear elements (LINE and SINE) are not enriched. The time of the burst of LTRs is later than the divergence time between the two species*.* Histograms showing the age distribution of LTRs located on the corresponding genome. Red lines indicate the divergence time between the two species. The Y axis shows the LTR content (bp) of the corresponding genome. A. LTRs are enriched within *C. croceus* and *A. crataegi* synteny breakpoint regions in the *A. crataegi* genome. The time of the burst of LTRs on *A. crataegi* genome is later than the divergence time between *A. crataegi* and *C. croceus.* B. LTRs are enriched within *C. croceus* and *P. brassicae* synteny breakpoint regions in the *A. crataegi* genome. The time of the burst of LTRs on *P. brassicae* genome is later than the divergence time between *P. brassicae* and *C. croceus.* C. LTRs are enriched within *C. croceus* and *P. rapae* synteny breakpoint regions in the *A. crataegi* genome. The time of the burst of LTRs on *P. rapae* genome is later than the divergence time between *P. rapae* and *C. croceus.* **Fig. S3.** Synteny between Wolbachia and two W chromosomes. There were six synteny blocks between Wolbachia and the W chromosome of *L. populi*. There were five synteny blocks between Wolbachia and the W chromosome of *L. camilla*. **Fig. S4.** Expression of *Geminin* in three species. A. Expression level of two *Geminins* in Monarch butterfly. The average FPKM of biological repeats is shown in the figures. B. Expression level of *Geminin* in Fall armyworm. The average FPKM of biological repeats is shown in the figures. C. Analysis of transcription levels of *Geminin* in silkworm by qRT-PCR. Data are shown as means ± SD of three experiments (**P < 0.01).

## Data Availability

The source of genome assemblies and transcriptome data supporting the analyses in this study are included in Additional file 1: Tables S1 and S9. The detailed information on ancestral chromosomes is included in Additional file 1: Table S2. All the gene sets annotated and analyzed in this paper have been deposited in the InsectBase 2.0 [5] (http://v2.insect-genome.com/Lep_anno). All data generated or analyzed during this study are included in this published article, its Additional files, and publicly available repositories.

## Abbreviations

Mya: Million years ago
RC: Rolling-circle Helitron transposon
LTR: Long terminal repeat
LINE: Long interspersed nuclear element
SINE: Short interspersed nuclear element
CARs: Continuous ancestral regions
FPKM: Fragments per kilobase of transcript per million fragments mapped
